# Novas Perspectivas no Tratamento da Hipertensão

**DOI:** 10.36660/abc.20201164

**Published:** 2021-03-03

**Affiliations:** 1 Universidade de São Paulo Instituto do Coração – Cardiopneumologia São PauloSP Brasil Universidade de São Paulo Instituto do Coração – Cardiopneumologia, São Paulo, SP – Brasil.

**Keywords:** Hipertensão/tendências, Pressão Arterial, Diuréticos/uso terapêutico; Doenças Cardiovasculares/complicações, Tratamento Farmacológico/tendências

A hipertensão arterial é certamente o maior fator de risco para doenças cardiovasculares. Tiocianatos, barbitúricos, brometos e bismuto foram testados no tratamento da hipertensão arterial no início da década de 1940.^1^ O uso dessas drogas foi interrompido por se mostrarem ineficazes e com diversos efeitos colaterais. Em meados da década de 1950, bloqueadores ganglionares como hexametônio, pentolínio, mecamilamina e substâncias simpatolíticas de ação periférica (guanetidina) foram testadas no tratamento da hipertensão arterial e mostraram-se eficazes na sua redução, mas pouco toleradas.^2^ Novos medicamentos foram introduzidos para este fim na década de 1950, incluindo diuréticos.^3^

Em 1956, em estudo observacional, Moser e Magaulay acompanharam 106 pacientes hipertensos que receberam rauwolfia, hidralazina, reserpina, mecamilamina e clorotiazida como tratamento para hipertensão arterial, isoladamente ou em combinação. A dose de clorotiazida utilizada neste estudo variou de 0,5 a 1,5 gramas e a combinação de clorotiazida resultou em melhor controle da pressão arterial. Desde esse estudo observacional até os dias atuais, vários estudos relacionados ao tratamento farmacológico da hipertensão foram realizados.^4^ O primeiro estudo randomizado e controlado por placebo realizado foi o *Veteran Administration* (VA), publicado em 1967.^5^ É importante notar que o critério de inclusão do estudo para o tratamento ativo *versus* placebo foi a pressão diastólica entre 115 e 129 mmHg. Após a publicação do estudo VA-I em 1967^5^ e do VA-II em 1970^6^, diversos estudos randomizados controlados abordando o tratamento da hipertensão arterial foram realizados. O foco inicial do tratamento para hipertensão arterial foi a excreção renal de sódio, uma vez que se pensava inicialmente que as doenças renais fossem a principal causa da hipertensão. Posteriormente, os mecanismos fisiopatológicos da hipertensão foram elucidados e a terapia foi direcionada aos principais deles. A ativação do sistema nervoso simpático como importante mecanismo fisiopatológico da hipertensão já havia sido percebida na década de 1950, e várias formas de bloqueio do sistema nervoso simpático e até simpatectomia foram então testadas.^7^ A partir da década de 1980, com a introdução da microneurografia e a técnica de spillover de norepinefrina, a importância da ativação do sistema nervoso simpático na fisiopatologia da hipertensão tornou-se ainda mais evidente.^8^^,^^9^ Embora mal tolerados, os bloqueadores adrenérgicos centrais e periféricos sempre fizeram parte do tratamento da hipertensão arterial.^10^ A ativação do sistema renina-angiotensina aldosterona (SRA) e a curva de pressão da natriurese alterada são dois outros mecanismos importantes na fisiopatologia da hipertensão arterial. Atualmente, o uso de diuréticos para correção da curva alterada de pressão da natriurese e de inibidores do sistema renina-angiotensina aldosterona, que atuam em diferentes locais, tem sido rotina no tratamento da hipertensão arterial.^11^

O sistema nervoso simpático e o sistema renina-angiotensina aldosterona estão envolvidos diretamente nos mecanismos fisiopatológicos da hipertensão arterial. Um aspecto importante nesse sentido é que a ativação desses dois sistemas está relacionada a um processo inflamatório no paciente hipertenso.^12^ Eles interagem com citocinas inflamatórias, como a interleucina-6 (IL-6) e o fator de necrose tumoral alfa (TNF-α). O sistema nervoso simpático estimula a secreção de citocinas pró-inflamatórias e, ao mesmo tempo, funciona como fonte de citocinas.^13^ A angiotensina-II é um importante fator pró-inflamatório. Está relacionada à produção de TNF-α e IL-6 e estimula a proteína-1 quimioatraente de monócitos (MCP-1) e o fator nuclear B.^14^^,^^15^ Por outro lado, o tipo de droga utilizada no tratamento de pacientes hipertensos pode reduzir o processo inflamatório relacionado à hipertensão. O uso de moxonidina simpatolítica de ação central no tratamento de mulheres hipertensas na pós-menopausa resultou em uma redução do TNF-α.^16^

Em um estudo envolvendo pacientes com hipertensão resistente, Barbaro et al. encontraram valores mais elevados de TNF-α em pacientes com hipertensão resistente em comparação com o grupo normotenso.^17^ Os resultados apontam para TNF-α como um possível mediador de dano vascular em pacientes com hipertensão resistente. Bautista et al.,^18^ encontraram associação dos níveis de IL-6 e TNF-α com os valores da pressão arterial, independentemente da idade, sexo, índice de massa corporal, história familiar de hipertensão e outras citocinas pró-inflamatórias. No estudo,^19^ os autores avaliaram o infliximabe, medicamento inibidor do TNF-α, para o tratamento de hipertensos resistentes. O infliximabe em dose única de 3 mg/kg (infusão) resultou em queda de 6,3 mmHg na pressão arterial média e 4,9 mmHg na pressão diastólica. Este efeito agudo do infliximabe na pressão arterial abre uma nova perspectiva no tratamento da hipertensão arterial. Independentemente da queda da pressão arterial, o uso de anti-TNF-α bloqueia um fator que pode ser agravante nas lesões vasculares. Sabe-se que a gravidade da hipertensão está relacionada a um ciclo vicioso decorrente da ativação dos sistemas pressóricos (sistema nervoso simpático e sistema renina-angiotensina-aldosterona).^20^ A interrupção desse ciclo é essencial para a proteção do organismo e para uma melhor ação de drogas hipotensoras já bem conhecidas.
