# Supplementary material for: Novas Perspectivas no Tratamento da Hipertensão
Source: Arq Bras Cardiol. 2021 Mar 3;116(3):452–3. [Article in Portuguese] doi: 10.36660/abc.20201164 (PMC8159559; doi:10.36660/abc.20201164)
Supplement: Translation

# Translation: New Perspectives in the Treatment of Hypertension

**Affiliations:** 1 São PauloSP Brazil Universidade de São Paulo Instituto do Coração – Cardiopneumologia, São Paulo, SP – Brazil.

**Keywords:** Hypertension/trends, Blood Pressure, Diuretics/therapeutic use, Cardiovascular Diseases/complications, Drug Therapy/trends

Arterial hypertension is by far the biggest risk factor for cardiovascular diseases. Thiocyanates, barbiturates, bromides and bismuth were tested in the treatment of arterial hypertension in the early 1940s.^1^ The use of these drugs was discontinued because they were proven ineffective and had several side effects. In the mid-1950s, ganglion blockers such as hexamethonium, pentolinium, mecamylamine and peripherally acting sympatholytic substances (guanethidine) were tested as treatment of arterial hypertension and were shown effective in reducing blood pressure, but little tolerated.^2^ New drugs were introduced to treat hypertension in the 1950s, including diuretics.^3^

In 1956, in an observational study, Moser and Magaulay followed up 106 hypertensive patients who received rauwolfia, hydralazine, reserpine, mecamylamine and chlorothiazide as treatment for arterial hypertension, alone or in combination. The dose of chlorothiazide used in this study ranged from 0.5 to 1.5 grams and the combination of chlorothiazide resulted in better control of blood pressure. Since that observational study by Moser and Magaulay to the present day, several studies related to the pharmacological treatment of hypertension have been carried out.^4^ The first randomized, placebo-controlled study conducted on the treatment of arterial hypertension was the Veterans Administration (VA), published in 1967.^5^ It is worth noting that the study inclusion criterion for active versus placebo treatment was diastolic pressure between 115 and 129 mmHg. After the publication of the VA-I study in 1967^5^ and the VA-II in 1970,^6^ several randomized controlled studies addressing the treatment of arterial hypertension were carried out. The initial focus of treatment for arterial hypertension was renal sodium excretion, as renal disorders were initially thought to be the main cause of hypertension. Subsequently, the pathophysiological mechanisms of hypertension were elucidated and the therapy was directed to the main pathophysiological mechanisms. The activation of the sympathetic nervous system as an important pathophysiological mechanism of hypertension had already been perceived in the 1950s, and various forms of sympathetic nervous system block and even sympathectomy were then attempted.^7^ Beginning in the 1980s, with the introduction of the microneurography and the norepinephrine spillover technique, the importance of activation of the sympathetic nervous system in the pathophysiology of hypertension was even more evident.^8^^,^^9^ Although poorly tolerated, central and peripheral adrenergic blockers have always been part of the treatment of arterial hypertension.^10^ The activation of the renin-angiotensin aldosterone system (RAS) and the altered natriuresis pressure curve are two other important mechanisms in the pathophysiology of arterial hypertension. Currently, the use of diuretics to correct the altered natriuresis pressure curve and inhibitors of the renin-angiotensin aldosterone system, which act at different sites, has been routine in the treatment of arterial hypertension.^11^

The sympathetic nervous system and the renin-angiotensin aldosterone system are involved directly in the pathophysiological mechanisms of arterial hypertension. An important aspect in this sense is that the activation of these two systems is related to an inflammatory process in the hypertensive patient.^12^ They interact with inflammatory cytokines such as interleukin-6 (IL-6) and the tumor necrosis factor-alpha (TNF-α). The sympathetic nervous system stimulates the secretion of proinflammatory cytokines and at the same time it functions as a source of cytokines.^13^ Angiotensin-II is an important proinflammatory factor. It is related to the production of TNF-α and IL-6 and stimulates the monocyte chemoattractant protein-1 (MCP-1) and the nuclear factor-B.^14^^,^^15^ On the other hand, the type of drug used in the treatment of hypertensive patients can reduce the inflammatory process related to hypertension. The use of centrally acting sympatholytic moxonidine in the treatment of postmenopausal hypertensive women resulted in a reduction in TNF-α.^16^

In a study involving patients with resistant hypertension, Barbaro et al.^17^ found higher TNF-α values in patients with resistant hypertension compared to the normotensive group.^17^ The results point to TNF-α as a possible mediator of vascular damage in patients with resistant hypertension. Bautista et al.^18^ found an association of IL-6 and TNF-α levels with blood pressure values, regardless of age, sex, body mass index, family history of hypertension and other proinflammatory cytokines. In the study,^19^ the authors evaluated Infliximab, a drug that inhibits TNF-α, for the treatment of resistant hypertensive patients. Infliximab in a single dose of 3 mg/kg (infusion) resulted in a drop of 6.3 mmHg in mean arterial pressure and 4.9 mmHg in diastolic pressure. This acute effect of Infliximab on blood pressure opens a new perspective in the treatment of arterial hypertension. Regardless of the drop in blood pressure, using anti-TNF-α blocks a factor that can be aggravating in vascular injuries. It is known that the severity of hypertension is related to a vicious circle resulting from the activation of pressure systems (sympathetic nervous system and renin-angiotensin aldosterone system).^20^ The interruption of this circle is essential to protect the organism and for a better action of hypotensive drugs already well known.
